# Experimental and formula deduction on the mechanical performance of *Fang* in *Dou-Gong* of Song dynasty

**DOI:** 10.1038/s41598-024-81623-4

**Published:** 2024-12-28

**Authors:** Long Zhang, Chuang Liu, Ting Zhou, Yuqing Qin, Song Gao

**Affiliations:** 1 School of Architecture, Tianjin University, Tianjin, 300072 China; 2 Tianjin International Engineering Institute, Tianjin University, Tianjin, 300072 China

**Keywords:** *Dou-Gong*, Quasi-static test, Theoretical analysis, Rigidity formula, Civil engineering, Nonlinear phenomena

## Abstract

In Song dynasty, *Dou-Gong* construction techniques, *Tou-Xin-Zao* and *Ji-Xin-Zao*, varied by the number of *Fang* connecting to the exterior. This study examines the impact of *Fang* connections on the mechanical characteristics of *Dou-Gong.* Six full-scale models were constructed and subjected to quasi-static loading tests in the horizontal Beam and *Fang* directions under vertical load. The hysteresis behavior, deformation, and stiffness variations were obtained and analyzed. The test results revealed the hysteresis curve of *Dou-Gong* developed into a flat shape, with good deformation recovery ability and seismic performance. Beam-direction loading led to brittle failure, with *Dou-Gong* having fewer *Fang* experiencing bearing capacity loss and those with more *Fang* succumbing to overturning. Beam-direction stiffness rose by approximately 29% as the number of connecting *Fang* increased. *Fang*-direction loading induced ductile failure, predominantly characterized by overturning. Notably, *Fang*-direction stiffness remained largely unchanged by the varying number of connecting *Fang*. *Dou-Gong* slip deformation ratio decreased by 5 -10% as the number of *Fang* increased. Furthermore, *Fang*-direction exhibited about 10% greater slip deformation capacity than the Beam-direction. Based on the force transfer mechanism of *Dou-Gong* components, a stiffness formula for the elastic stage of *Dou-Gong* in the Beam-direction and *Fang*-direction was established and validated against experimental data.

## Introduction

**Fig. 1:**
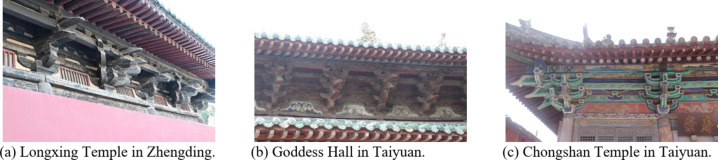
Partial Song Dynasty’s *Dou-Gong* of China.

*Dou-Gong*, a pivotal and essential element in ancient Chinese architecture and cultural heritage. In particular, the Song dynasty’s *Dou-Gong*, as shown in Fig. 1, is the most emblematic, exhibiting comprehensive structure and function, and is crucial for both decoration and support in wooden architecture^1^.

**Fig. 2:**
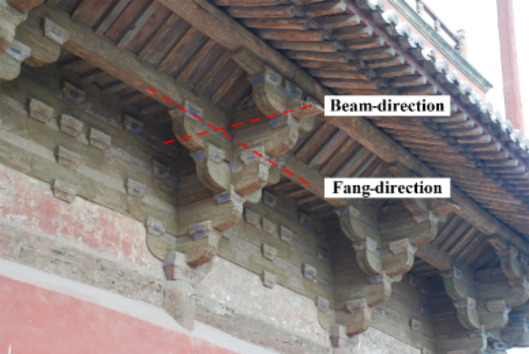
The main horizontal force direction of *Dou-Gong*.

**Fig. 3:**
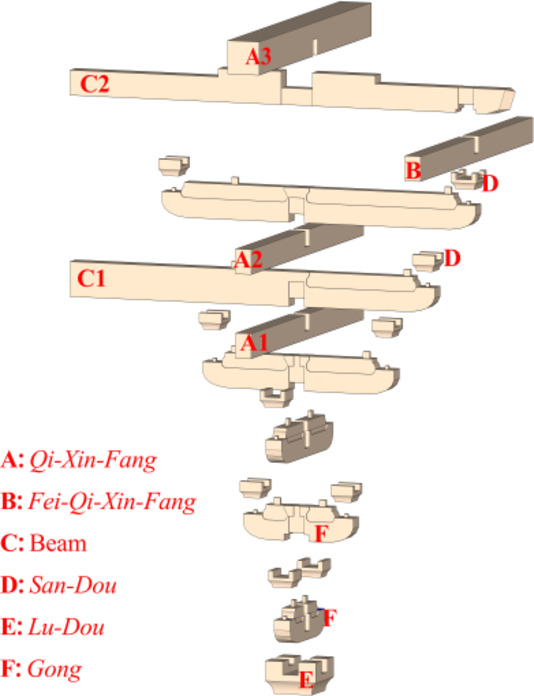
The composition diagram of Dou-Gong in Song dynasty.

The two horizontal force directions of *Dou-Gong* mentioned in this paper are Beam-direction and *Fang*-direction, which are orthogonal as shown in Fig. 2. *Dou-Gong* is composed of several longitudinal and transverse beams (*Gong*) and support blocks (*Dou*) as shown in Fig. 3. The *Lu-Dou* component connects with the column, and the *Fang* or beam component connects with *Gong*. The small blocks between components are called *Dou*, which are set on both ends to support an upper layer of *Gong*s^2^.

For Fig. 3, a detailed description of each wood component is presented in Table 1, where L is the component length, and where B and H denote the section width and height, respectively.

**Table 1:** Component identification and the corresponding dimensions (unit: mm).

No. (From bottom to top)	Name	L×B×H (Integral components)	B×H (Contact local components)
A1 / A2	*Qi-Xin-Fang*	2000 × 100 × 150	100 × 50
A3	*Qi-Xin-Fang*	2000 × 200 × 200	200 × 100
B	*Fei-Qi-Xin-Fang*	2000 × 100 × 150	100 × 50
C1	Beam	2327 × 100 × 210	100 × 160
C2	Beam	2825 × 100 × 150	100 × 100
D	*San-Dou*	Top:160 × 140 × 60 Bottom:120 × 100 × 40	-
E	*Lu-Dou*	Top:320 × 320 × 120 Bottom:240 × 240 × 80	-

At present, more studies have focused on *Dou-Gong* nodes, not only on Chinese *Dou-Gong*, but also the foreign ones^3–6^, and hysteresis curves have been obtained through experiments to assess the energy dissipation capacity in seismic action^7–9^. With respect to earthquake performance, many researchers have conducted experiments to explore the seismic resistance capacity^10^. For example, when quasistatic tests are conducted, a hysteresis curve is obtained^11–13^. In addition, several dynamic tests have been performed^14–16^, for example, the researcher^17^ studied the dynamic performance with a 1:5 scaled Pagoda model under various excitation intensities, only slightly damages occurred in the earthquake, which demonstrated its good earthquake resistance. When *Dou-Gong*is subjected the vertical load, the friction between the components can enhance the stiffness and energy dissipation capability, in^18^, it presented experiments on the eccentrically aligned *Dou-Gong*. In^19,20^, it was found that the lateral stiffness also enlarges with the increment of the vertical load. With numerical analysis^21,22^, the results of the experiments could be verified, and the skeleton curves were in good agreement with the experimental results^22–24^. Recently, studies have concentrated on the internal components of joints. For instance, the effects of *Fang* or *Ang*on the mechanical properties of the joints had been investigated^23,25^. Some researchers started to focus on the study of the differences in mechanical properties of Dou-gong under loading in *Fang*-direction and Beam-direction. These studies compared the bearing capacity in two directions tests and obtained that the stiffness in Beam-direction is greater than that in *Fang*-direction^26–29^. In addition, the researchers explored the different relationship between the positive and negative loading capacities for different directions of loading by numerical analysis^30,31^. However, there were few studies on the effects of internal components for the mechanics of the whole joints such as *Fang*. To address this problem, the following research is carried out in this paper.

**Fig. 4:**
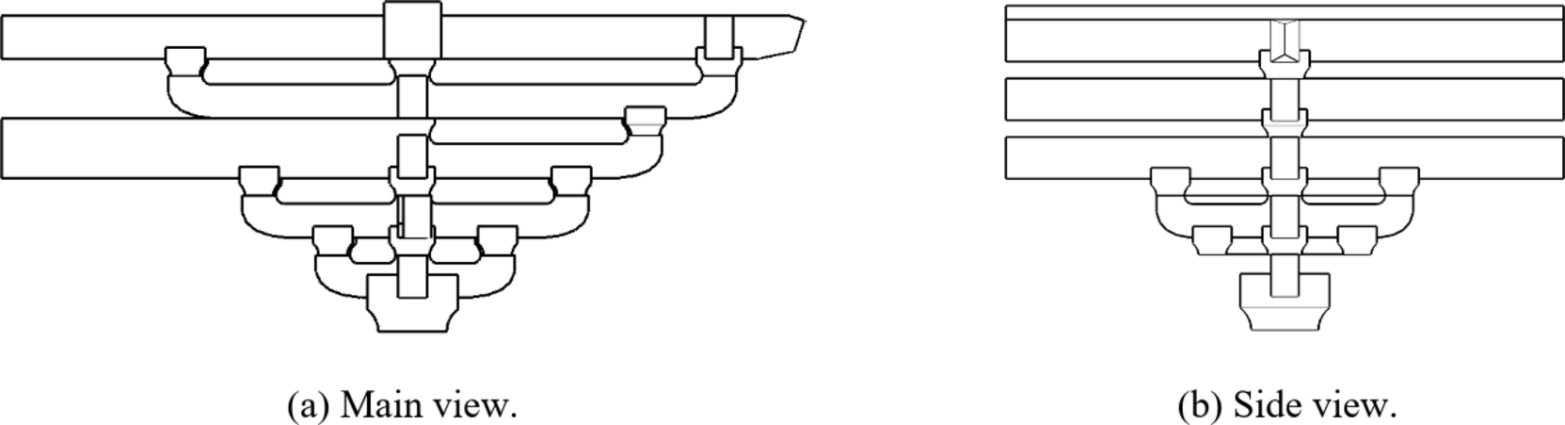
Completely *Tou-Xin-Zao Dou-Gong*.

**Fig. 5:**
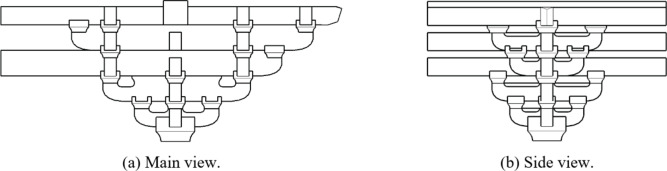
Incompletely *Tou-Xin-Zao Dou-Gong*.

**Fig. 6:**
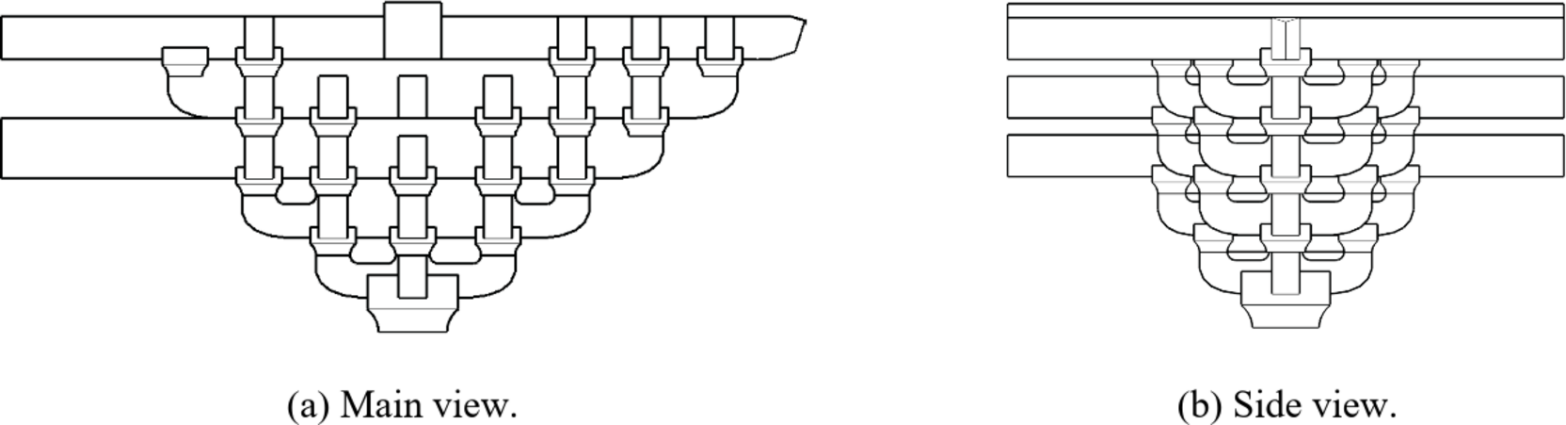
Ji-Xin-Zao Dou-Gong.

In *Tou-Xin-Zao Dou-Gong*, only part of the beam components set *Fang* components and *Gong* components. As shown in Fig. 4, there is no *Fang* component or *Gong* component set on the end of each beam component, which is called completely *Tou-Xin-Zao Dou-Gong*. As shown in Fig. 4, the first and second beam components don’t include the *Fang* or *Gong* component. This kind of *Dou-Gong* called incompletely *Tou-Xin-Zao Dou-Gong*. From the perspective of mechanics, *Dou-Gong* is connected with the external structure by only one *Fei-Qi-Xin-Fang* component in Fig. 5, but is connected by six *Fei-Qi-Xin-Fang* components in Fig. 6, which is called *Ji-Xin-Zao Dou-Gong*. Therefore, several distinctions may exist between these two joints and will be studied in this paper. When it is used for the overall structure simulation, the spring is used to simulate the joint, so it is necessary to know the stiffness value in each force direction. The main purpose of this paper is to explore the differences between *Dou-Gong* with different numbers of *Fei-Qi-Xin-Fang* and deduce the stiffness calculation formula.

## Test program

### Test model

**Fig. 7:**
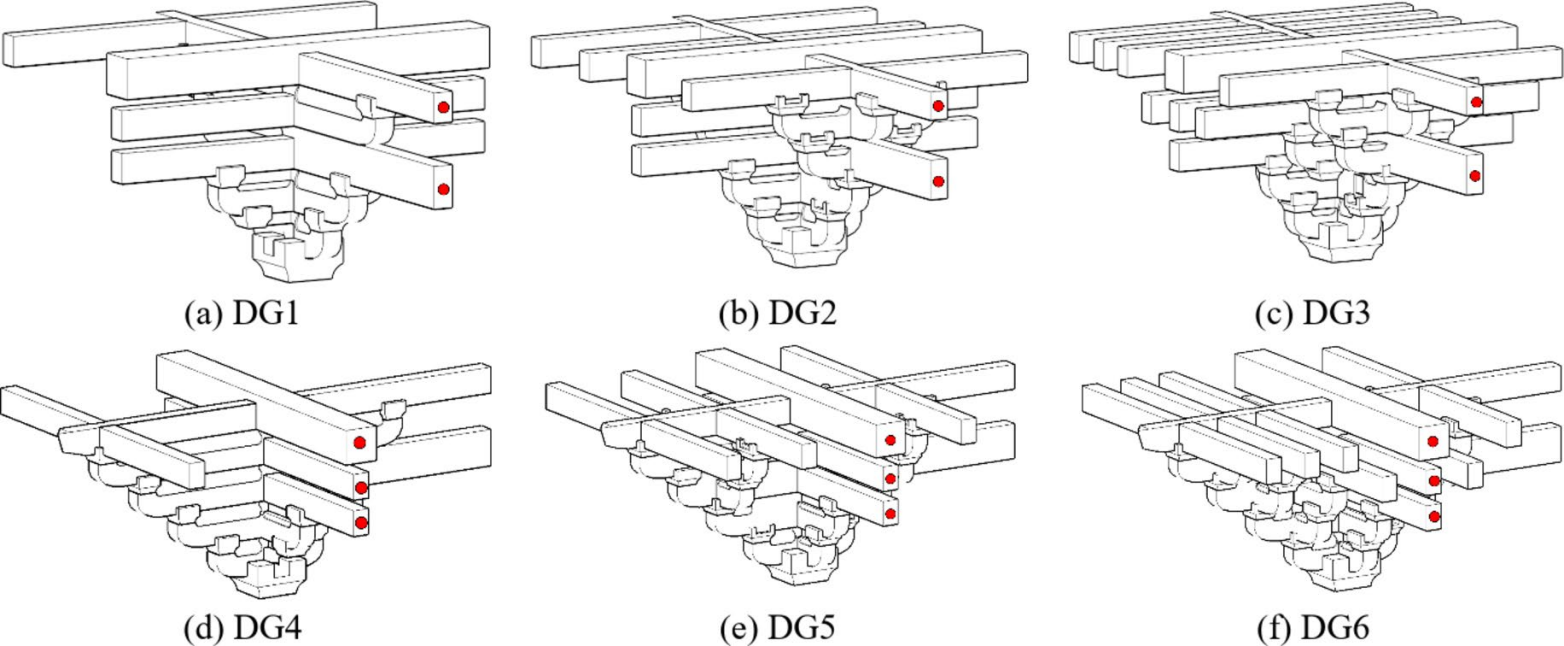
Three-dimensional model with loading points.

To study the influence of different techniques (the number of connected *Fang*) on the mechanical properties of *Dou-Gong*, according to ***Ying Zao Fa Shi***(Treatise on Architectural Methods or State Building Standards)^32^, in this study, *Dou-Gong* was fabricated following the joint placed in Guanyin Pavilion Dule temple Jixian Country as the incompletely *Tou-Xin-Zao Dou-Gong* termed as DG2/DG5 in Fig. 5. By changing the number of *Fang*, a set of completely *Tou-Xin-Zao Dou-Gong* (DG1/DG4) and a set of *Ji-Xin-Zao Dou-Gong* (DG3/DG6) were designed as shown in Figs. 5 and 6. Among them, DG1, DG2, and DG3 were utilized for studying loading in Beam-direction, whereas DG4, DG5, and DG6 were used to study loading in *Fang*-direction. And the loading points were indicated by red dots as illustrated in Fig. 7.

To ensure the restoration of *Dou-Gong* in ancient architecture, traditional hand tools were used throughout the assembly process. The growth characteristics determine the anisotropy of wood, so that the wood has three natural directions: the longitudinal direction along the extension direction of the trunk, which is referred to as L, the radial direction perpendicular to the annual rings from the center of the trunk outwards (R), and the chord direction consistent with the direction of the annual rings (T). Pinus sylvestris, which was widely used in ancient buildings, was chosen as the material for *Dou-Gong*model. The mechanical parameters were obtained by our research group through standard material testing^25,28,33^, and the mechanical properties were shown in Table 2.

**Table 2:** Mechanical properties of Pinus sylvestris.

Modulus	Explanation	MPa
$\:{E}_{L}$	L-direction compressive modulus of elasticity	2548.4
$\:{E}_{R}$	R-direction compressive modulus of elasticity	241.9
$\:{E}_{T}$	T-direction compressive modulus of elasticity	159.9
$\:{E}_{WR}$	R-direction bending modulus of elasticity	8153.5
$\:{E}_{WT}$	T-direction bending modulus of elasticity	7876.5

### Test setup and data measurement

In the normal working state, *Dou-Gong* was loaded in three directions. The vertical load was transformed by purlins and applied at the position right above *Lu-Dou* in upper surface of the upper Qi-Xin-*Fang*, where a jack weighing 30 tons was placed beneath a roller that could slide along the girder to provide a vertical sustained load during the process of horizontal loading to simulate the dead load of the rooftop.

**Fig. 8:**
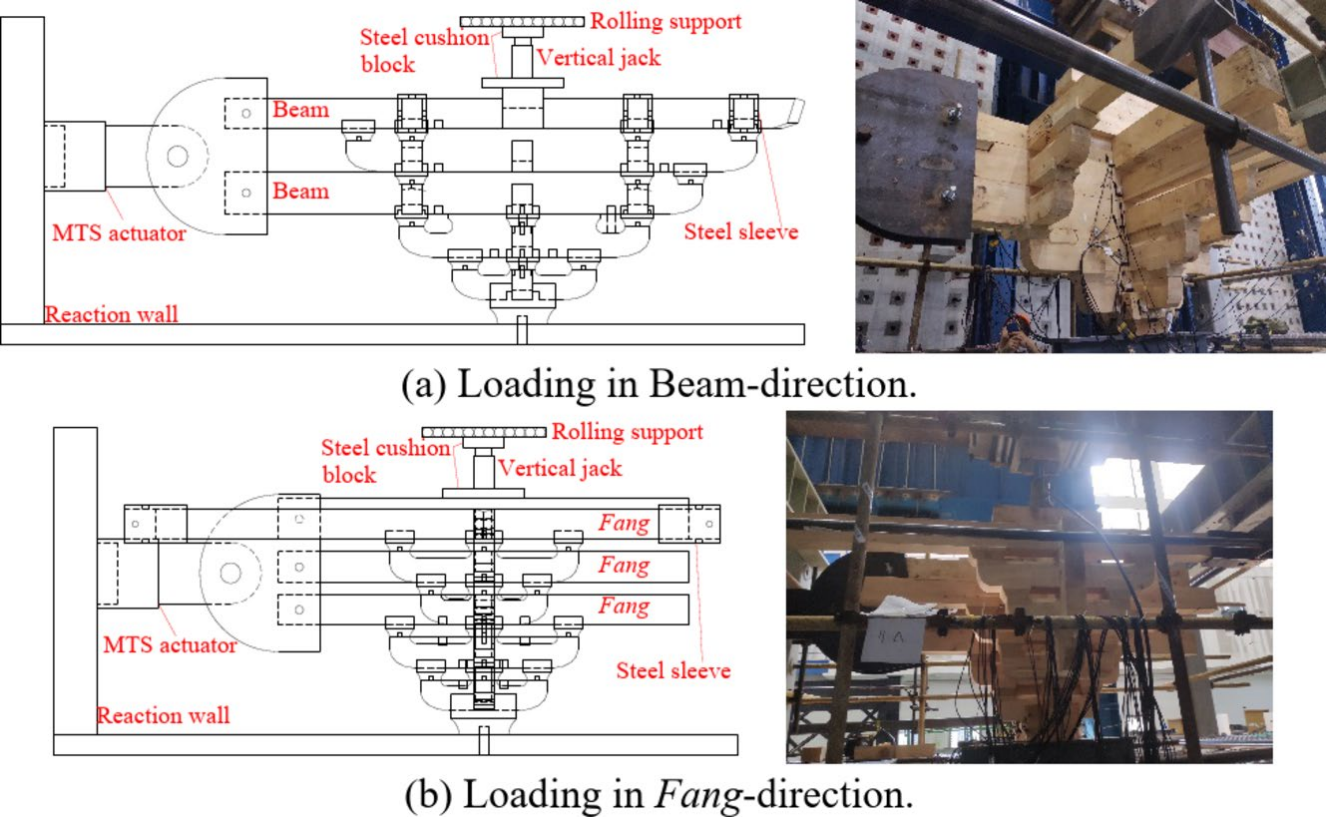
The quasi-static experimental device.

**Fig. 9:**
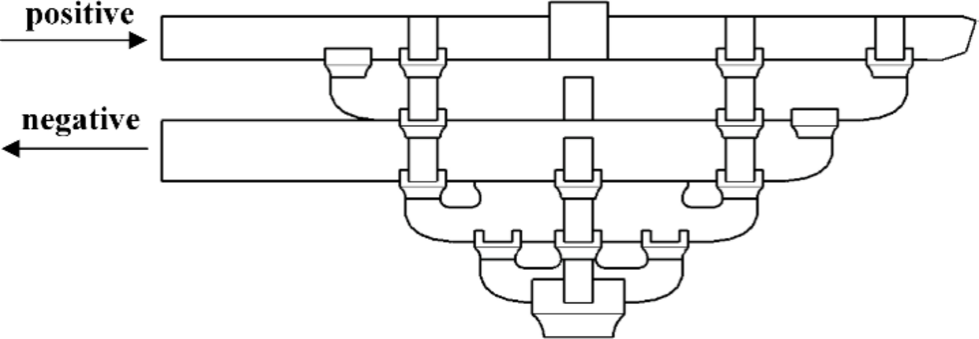
Force direction diagram of *Dou-Gong*.

Because *Dou-Gong* was not symmetrical, the horizontal cyclic load was applied in two different directions. In Beam-direction, the horizontal cyclic load was applied to the end of the Beam in the first group by a MTS actuator, which could collect load and displacement data simultaneously as shown in Fig. 8(a). Additionally, the actuator produces a horizontal reciprocating motion, the positive direction was referred as the joint under the pushed condition and the displacement was recorded as ‘+’, and the negative was for the pulled condition and the displacement was recorded as ‘-’ shown in Fig. 9. In the direction of *Fang*, the horizontal cyclic load was applied to the end of the *Fang* as shown in Fig. 8(b).

**Fig. 10:**
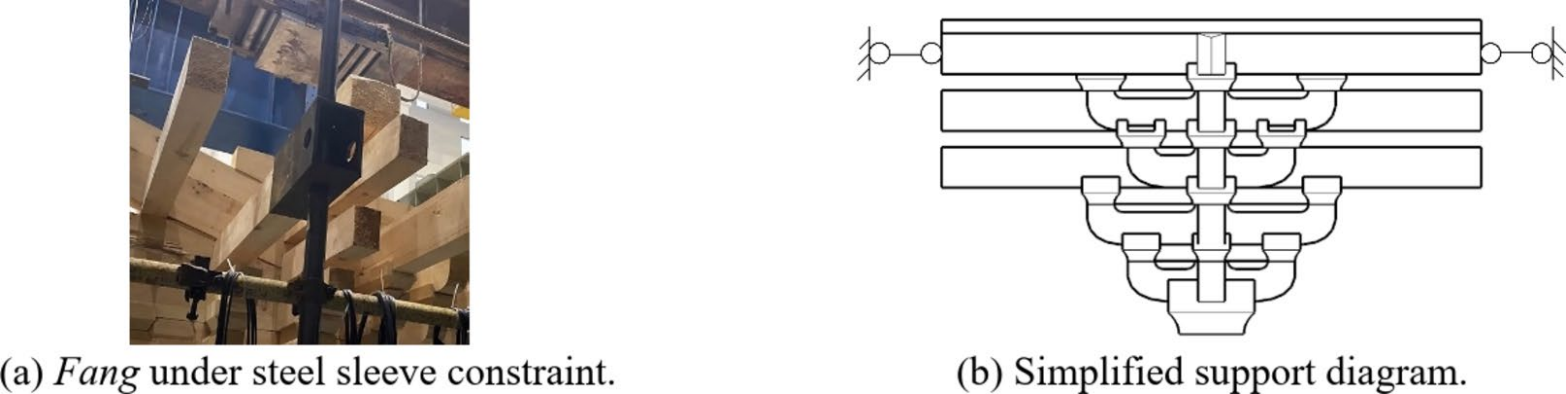
The articulated device of *Fei-Qi-Xin-Fang*.

The midpoint of the connected *Fang* was the point of inflection in reality; hence the *Fei-Qi-Xin-Fang* components were fixed on the counter-force frame through articulation by the devices to assure the actual degrees of freedom, as shown in Fig. 10.

**Fig. 11:**
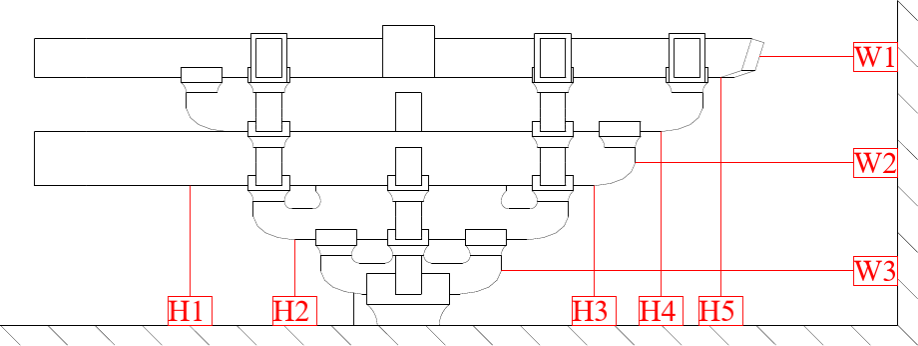
Displacement sensor layout diagram.

The displacement transducer layout diagram during loading was shown in Fig. 11, with three linear variable displacement transducers used to gauge the vertical deformation, and the other five transducers used for the horizontal deformation. Due to the small horizontal displacement and the small overall rotation angle, the effect of vertical displacement on horizontal displacement was not considered in this study and will be further considered in subsequent tests. Thirty strain gauges were placed on the key positions of the components, including *Dou*, *Gong*, Beam and *Fang* components, to measure the strain during the loading procedure. All the data were recorded in real time via WKD3813 strain data acquisition instructions.

**Fig. 12:**
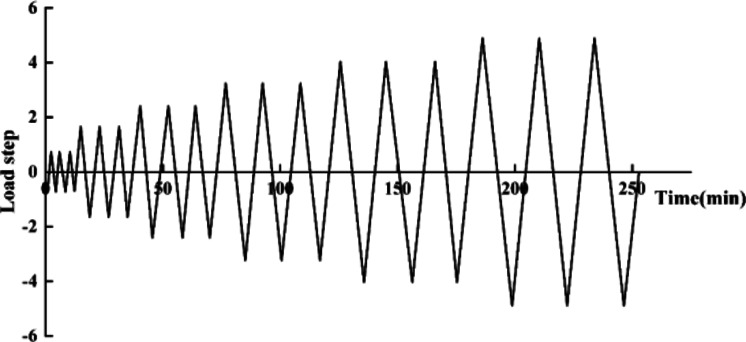
Loading scheme.

Considering that most of the damage of ancient buildings originates from displacement overrun, and MTS actuator was more convenient for controlling displacement, displacement control was chosen as the horizontal loading method. In this study, we referred to the roof load of ***Ying Zao Fa Shi***^32^ and calculated the vertical load transferred to *Dou-gong* according to ***Preliminary Investigation of the Static Force of Damuzuo***^34^, and obtained the vertical load is 50 kN^35,36^. First, a horizontal load of 10 kN was applied to the model, cycled for a period to ensure the proper function of the force sensor and measurement devices, and recorded the maximum displacement as a reference loading level after rounding. After the preloading was completed, a vertical load of 50 kN was applied to the model during the whole loading process, and the model was subjected to the low cyclic loading until it was destroyed^37^. The low-cycle reverse cyclic loading adopted the loading system shown in Fig. 12 was adopted, with each step being cycled three times.

## Phenomenon and analysis

### Experimental phenomenon

**Fig. 13:**
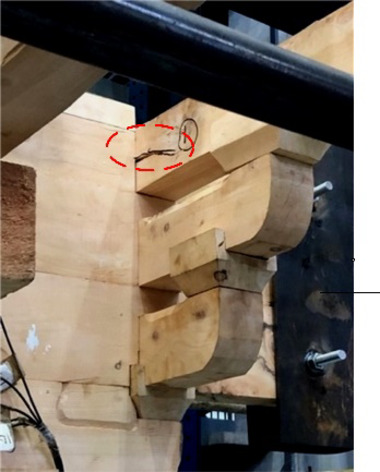
Specimen cracks.

**Fig. 14:**
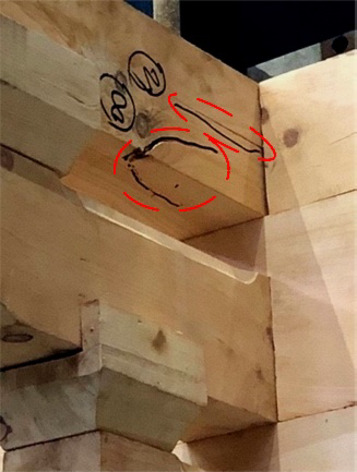
Developed cracks.

**Fig. 15:**
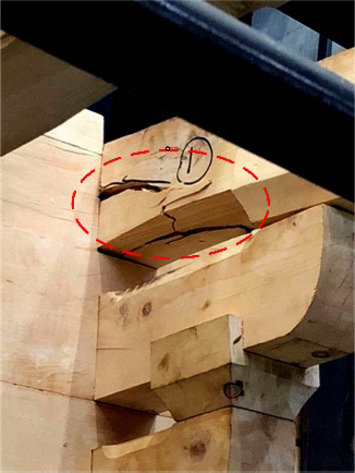
The end of *Fang* was fractured.

**Fig. 16:**
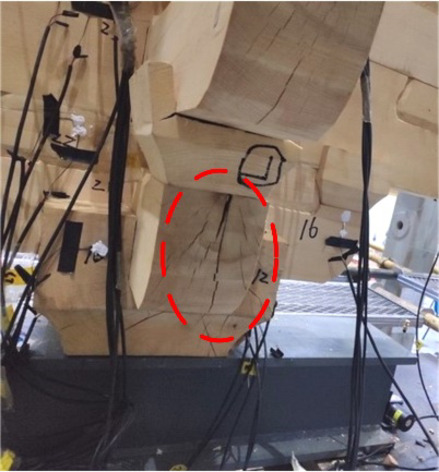
Dou-Er in *San-Dou* was damaged.

When loaded in Beam-direction, during the whole process of applying a vertical load, accompanied by the compression of the gap in the buckets, there was only a slight creaking sound and no cracks appeared. At the initial stage of horizontal low cyclic load application, the specimen creaked from time to time, which was caused by the friction between the wood surfaces of the components. At this time, part of the root of *Fang* components cracked on just one side of the members without penetration shown in Fig. 13. With the load increasing, the cracks expanded, along with the number of cracks growing simultaneously, and were concentrated in the joints of the beam and *Fang* components shown in Fig. 14. The damage could be found mainly at the end of the beam and *Fang* components, and the through cracks and fractures were the signs and causes of the final damage of the bucket shown in Fig. 15. In addition, the phenomenon of damage to the *Dou-Er* of the *San-Dou* was more common shown in Fig. 16, where *Dou-Er* is the protruding part on top of *Dou*. But the impact of the damage to the *Dou-Er* on the load-bearing capacity of the bucket was relatively tiny. The better integrity of *Lu-Dou* with cracks which did not develop significantly, indicating that the damage of the beam and *Fang* components under the horizontal load was prior to that of *Lu-Dou*. With the increase of the number of *Fang*, DG1 and DG2 lost the overall bearing capacity and stopped the test due to the fracture of *Fang* when the displacement was 60 mm and 80 mm, respectively. When the displacement was 100 mm in DG3, the overall structure was relatively intact despite the cracking and failure of *Fang*. Finally, the loading stopped because of excessive loading displacement and overall displacement.

However, when loaded in *Fang*-direction, the phenomenon was not the same as that of Beam-direction. During the whole loading process, there was no visible damage in DG4, DG5, DG6 and the experiment ends with the excessive displacement. Especially in DG5, it stopped loading due to the maximum slide travel of *Fang* in the test setup, since the *Fei-Qi-Xin-Fang* is constrained by the steel sleeve.

### Results and analysis

#### Hysteretic curve

**Fig. 17:**
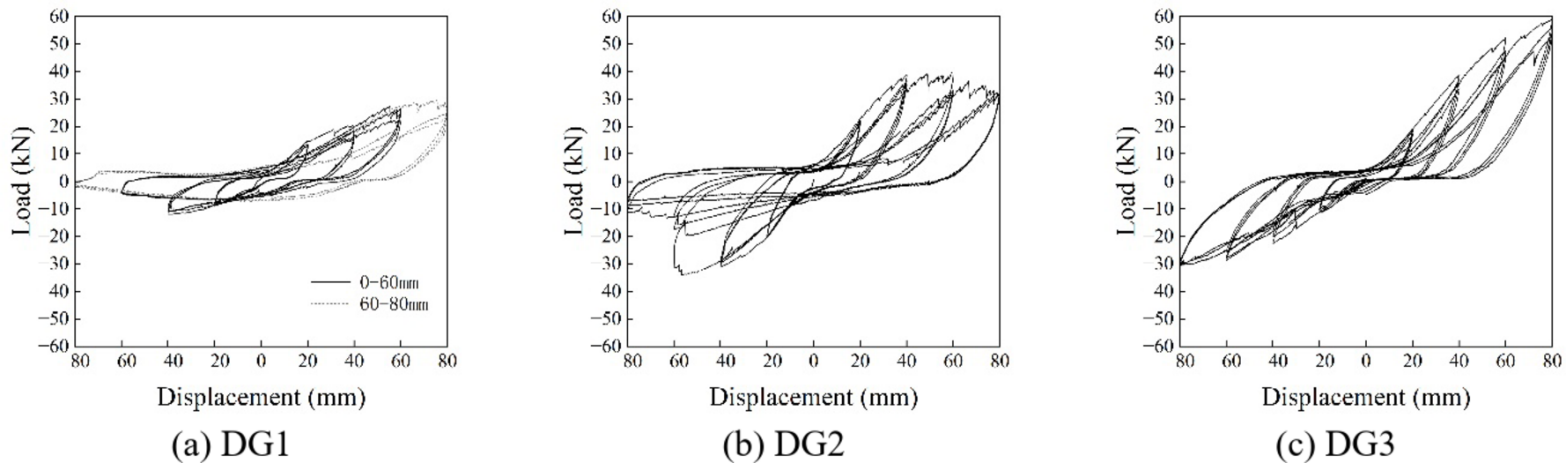
The hysteretic curve in Beam-direction.

**Fig. 18:**
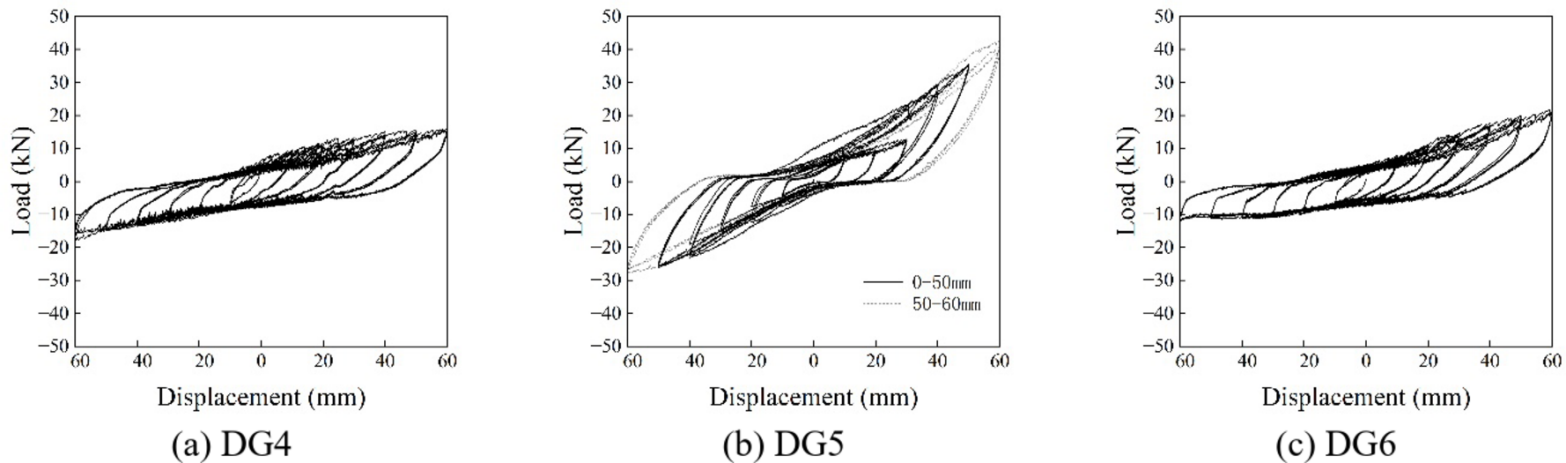
The hysteretic curve in *Fang*-direction.

The hysteretic curves obtained from the tests are given in Figs. 17 and 18, and the dashed line in DG1 represents the complete fracture of the only *Fang* that provided horizontal resistance, whereas the dashed line in DG5 represents the cessation of loading due to the maximum sliding stroke of *Fang*.

It appeared to exhibit a reverse S shape. At the initial stage of each loading cycle, the curve is relatively flat with a small slope, and increases significantly when it approaches the peak value, which indicates the horizontal displacement is provided mainly by the compression of the gaps between components. The deformation caused by components themselves is tiny, and so as the overall rigidity of *Dou-Gong*. After the gaps are fully compressed, the new horizontal displacement mainly comes from the deformation of the components themselves. Therefore, the overall rigidity increases with the slope increasing significantly. When the load is unloaded to a small extent, there is still a certain residual horizontal displacement for the gaps between the components are compressed, but cannot be recovered during unloading. The obvious horizontal segment of the curve is the process of *Dou-Gong* gaps developing in the opposite direction.

#### Skeleton curve

**Fig. 19:**
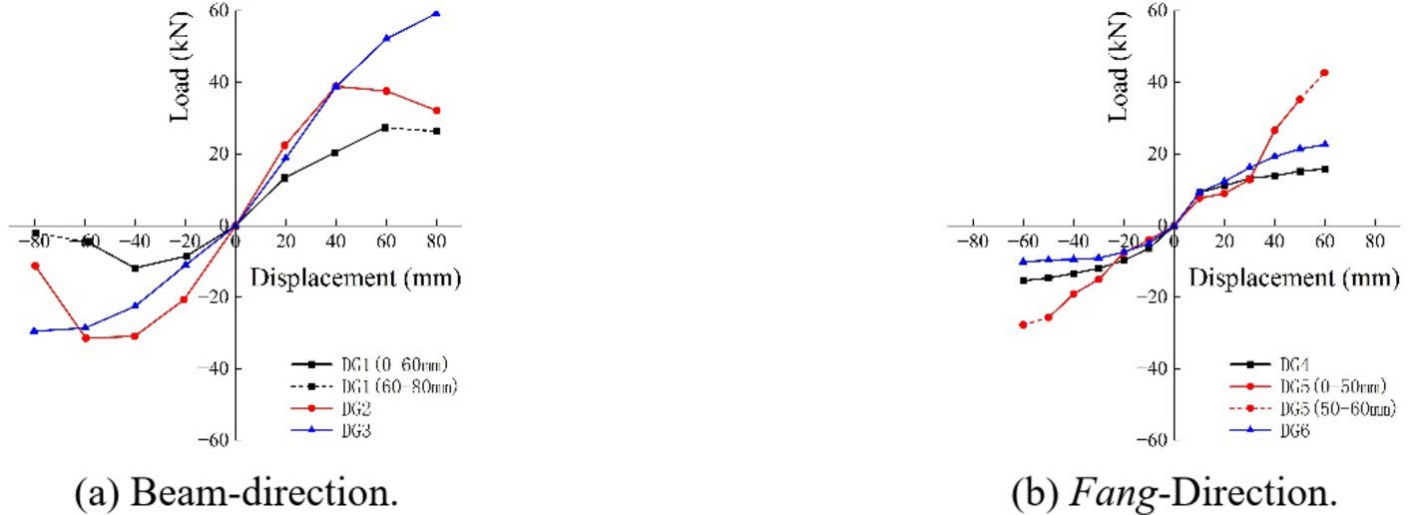
The skeleton curves.

The skeleton curves are given in Fig. 19. For a given loading amplitude Δ, each point in the skeleton curve is determined by the maximum displacement and the corresponding force at the first cycle of each loading stage of the hysteretic loop. Overall, the bearing capacity of *Dou-Gong* increases continuously with increasing horizontal displacement, and begins to decrease after the bracket is damaged.

In Beam-direction, DG1 and DG2 have a descent section after destruction. Figure 19(a) shows that the bearing capacity of *Dou-Gong* increases with increasing number of *Fang*. In *Fang*-direction, the trend is roughly the same as that in Beam-direction. When DG5 is loaded to the last two steps, the slide travel reaches the maximum, and the skeleton curve rises obviously with the bearing capacity increases. When the horizontal displacement is under 30 mm, the horizontal bearing capacity only have little difference of all joints. There is no obvious relationship between the horizontal bearing capacity and *Dou-Gong* technique.

#### Horizontal rigidity

**Fig. 20:**
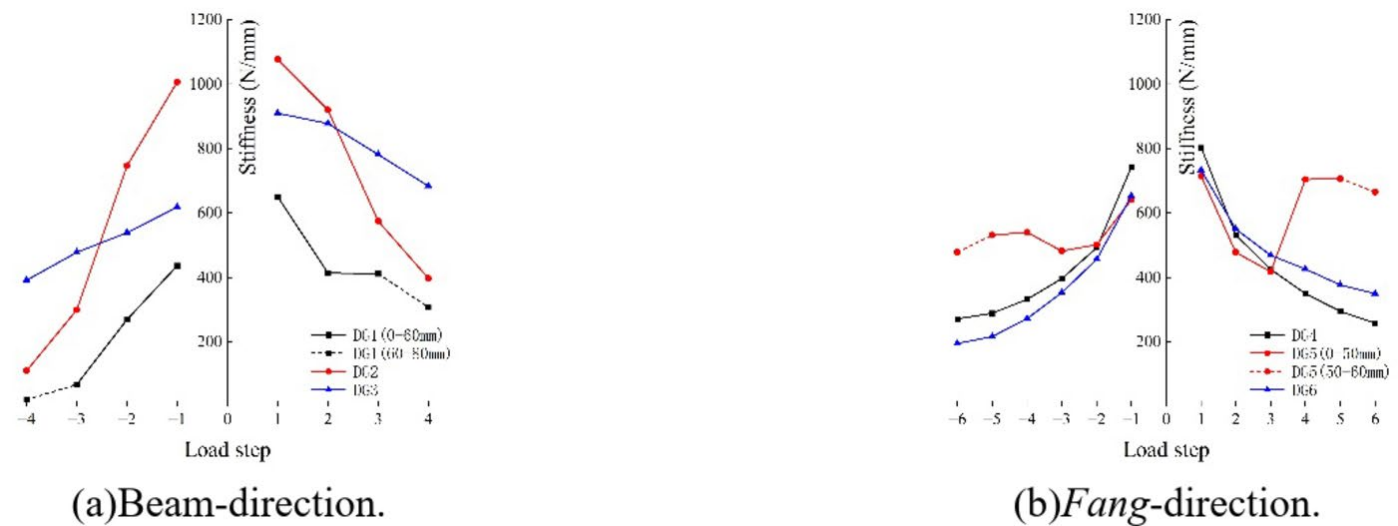
Stiffness degradation curve.

The horizontal rigidity$\:\:{k}_{i}$ of *Dou-Gong* can be calculated via Eq. (1), where *F*_*i*_ is the *i*-th load, *u*_*i*_ is the horizontal displacement peak corresponding to *F*_*i*_. The stiffness degradation curves are presented in Fig. 20. It is clear that the horizontal stiffness of all *Dou-Gong* decreased as load step increased.

1 $$\:{k}_{i}=\frac{{F}_{i}}{{u}_{i}}$$

In Beam-direction, the stiffness of *Dou-Gong* increases with the increase of the number of beams. In *Fang*-direction, the difference in stiffness between Dou-Gongs is not significant. The last two stages of sliding rail travel loaded by DG2 reach its maximum value, resulting in an upwards segment in the stiffness curve.

According to the data analysis, the stiffness values in the positive direction of each elastic stage of *Dou-Gong* are shown in Table 3.

**Table 3:** Stiffness comparison of different *Dou-Gong*.

Number of DG	DG1	DG2	DG3	DG4	DG5	DG6
Number of connecting *Fang*	1	3	5	1	3	5
Stiffness (N/mm)	412.1	575.0	781.3	532.2	477.7	550.8

Based on the stiffness data of three *Dou-Gong* loaded in Beam-direction, it can be concluded that the core of *Dou-Gong*(excluding *Fang*) provides a stiffness of approximately 320 N/mm, and for each additional *Fang*, the stiffness increases by 28.9%.

#### Strength degradation

The load peak of the first and last periods of each load level is used to calculate the strength degradation rate of each *Dou-Gong*.

**Fig. 21:**
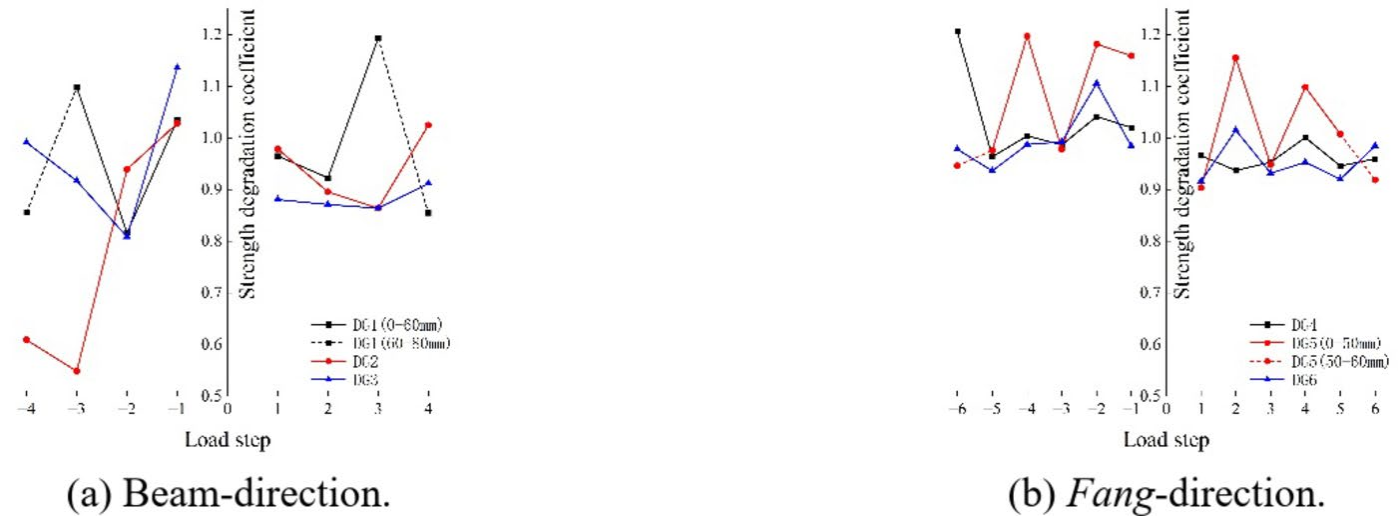
The strength degradation curve.

According to the strength degradation curve in Beam-direction shown in Fig. 21 (a), in the positive direction, the strength degradation coefficient is sometimes less than 0.9, indicating that the bearing capacity of *Dou-Gong* drops rapidly and that damage occurs suddenly, which is brittle failure in terms of damage type. In the negative direction, the factor is sometimes less than 0.6, so the bearing capacity of *Dou-Gong* decreases faster, leading to more sudden and obvious brittle damage. An analysis of the curves in the *Fang*-direction in Fig. 21 (b), reveals that when the coefficient at all load steps is greater than 0.9, the bearing capacity decreases slowly, and damage occurs slowly, which is ductile failure.

In addition, some loading stages have a strength degradation coefficient greater than 1, which is due to the existence of certain gaps in *Dou-Gong*, resulting in a lower initial bearing capacity. With the loading process, the strength undergoes strengthening.

#### Deformation ability

As the timber structure often does not exhibit a clear yield phenomenon before failure, a sliding deformation ratio *r* (the ratio of slip deformation $\:{d}_{p}$ to total deformation $\:d$) is defined to measure the deformation ability as shown in Eq. (2). One of the characteristics of the traditional wooden structure *Dou-Gong* is that the slip between components is non-destructive and can be recovered when subjected to reciprocating loads, whereas the deformation of a component is usually unrecoverable. Therefore, the greater the sliding and deformation capacity of *Dou-Gong* is, the better the deformation capacity of *Dou-Gong* under the action of an earthquake. Specifically, the larger the sliding deformation ratio is, the stronger the corresponding deformation ability of *Dou-Gong*.

2 $$\:r=\frac{{d}_{p}}{d}$$

Taking DG1 as an example, the residual deformation after unloading is 39.90 mm and the sliding deformation ratio is 0.665 under the load step of 60 mm in Beam-direction. Under the load step of 60 mm in *Fang*-direction, the residual deformation after unloading is 46.38 mm, and the slip deformation ratio is 0.773. By comparing the deformation ratio, the deformation ability of DG1 in *Fang*-direction is better than that in Beam-direction. According to Eq. (2), the specific slip deformation ratio data are shown in Table 4.

**Table 4:** Calculation of the slip deformation ratio of *Dou-Gong*.

Load step(mm)			20	−20	40	−40	60	−60	average
Beam-direction	DG1	slip deformation	13.05	−12.88	27.42	−27.47	41.53	−46.05	0.688
		ratio	0.653	0.644	0.686	0.687	0.692	0.768	
	DG2	slip deformation	11.86	−8.67	21.09	−16.95	38.26	−36.89	0.538
		ratio	0.593	0.434	0.527	0.424	0.638	0.615	
	DG3	slip deformation	10.95	−9.36	19.89	−21.96	31.71	−31.23	0.518
		ratio	0.548	0.468	0.497	0.549	0.529	0.521	
*Fang*-direction	DG1	slip deformation	12.58	−13.55	28.53	−28.35	43.09	−47.2	0.706
		ratio	0.629	0.678	0.713	0.709	0.718	0.787	
	DG2	slip deformation	12.87	−13.04	22.31	−25.98	/	/	0.626
		ratio	0.644	0.652	0.558	0.650	/	/	
	DG3	slip deformation	11.34	−11.78	20.95	−22.75	34.23	−42.62	0.588
		ratio	0.567	0.589	0.524	0.569	0.571	0.710	

According to the data in Table 4:.

(1) For a specimen loaded in one specific direction, the deformation ability of *Dou-Gong* increases with increasing load step, which is reflected in the increase of slip deformation ratio by about 5–10% for each increase of horizontal displacement.

(2) The deformation ratio decreases 5–10% with each increase of *Fang* number when loaded in the same direction.

(3) For the same joint, the deformability in *Fang*-direction is greater than that in Beam-direction. The sliding deformation in *Fang*-direction is about 10% higher than that in Beam-direction.

## Stiffness formulas of *Dou-Gong*

### Stiffness formula in Beam-direction

#### The stiffness provided by Fang

**Fig. 22:**
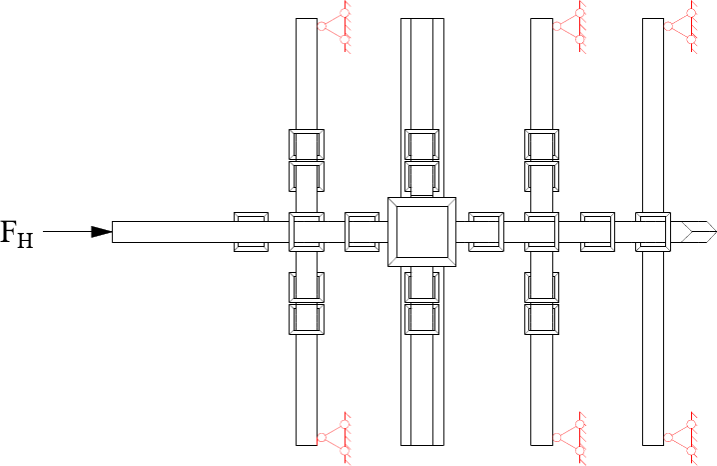
Bottom view of *Dou-Gong* under horizontal loading in Beam-direction.

As shown in Fig. 22, when *Dou-Gong* is under the load in Beam, it tends to displace along the loading direction. The constraints at both ends of the *Fang* limit the horizontal displacement of the *Fang* and resist the displacement trend, so part of the horizontal stiffness of *Dou-Gong* is provided by *Fang*.

**Fig. 23:**
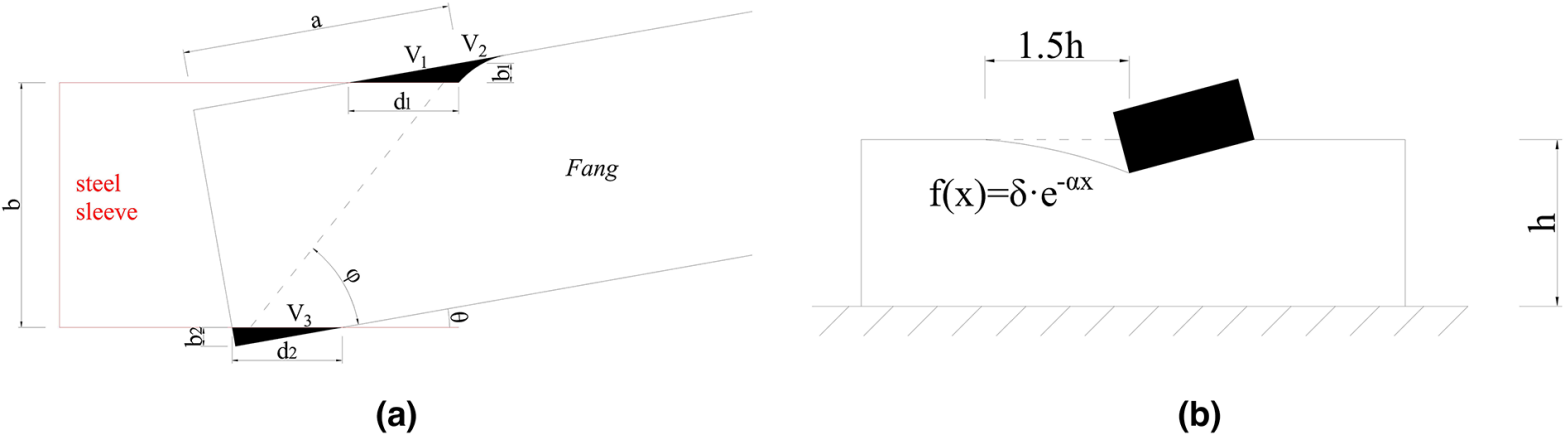
Embedded compression deformation diagram of *Fang*. (a) Compression diagram of Fang. (b) Mechanism of deformation in indirectly compressed areas^41^ .

According to Inayama M and Tanahashi H et al.^38–40^, the deformation of Fang when constrained at both ends is provided by a combination of bending and embedded compression deformation, where the embedded compression deformation is shown in Fig. 23.

(1) Embedded compression deformation stiffness.

In Fig. 23 (a), when the square is rotated at an angle $\:\theta\:$, an upper and lower part of the deformation zone is created due to the steel sleeve. The upper deformation zone consists of a direct deformation zone V1 and an indirect deformation zone V2, and the lower deformation zone consists of a direct deformation zone V3.

The zone of indirect deformation is generally expressed with an exponential curve as Eq. (3).

3 $$\:f\left(\text{x}\right)=\delta\cdot{e}^{-\alpha\:x}$$

where the horizontal length of the curve in Fig. 23 (b) is called the decay length and is assumed to be 1.5 h, h is the thickness of the pressed wood member, and $\:\delta\:$ is the direct force compression displacement. The shape of the exponential curve is determined by a coefficient $\:\alpha\:$, which takes the value of 1.5/h^40^.

The compression deformation $\:{\varDelta\:}_{c}$ is expressed as Eq. (4), where $\:{l}_{f}$ denotes the length of Fang*.*

4 $$\:{\varDelta\:}_{c}=\theta\cdot\left(\frac{{l}_{f}}{2}-a\right)$$

The geometric relationship in Fig. 23 (a) can be obtained as follows:

5 $$\:\text{Tan}\phi\:=\frac{b}{a}$$

6 $$\:{d}_{1}\bullet\:\text{tan}\theta\:+b+{d}_{2}\bullet\:\text{cos}\theta\:\bullet\:\text{sin}\theta\:=\sqrt{{a}^{2}+{b}^{2}}\bullet\:\text{sin}\left(\phi\:+\theta\:\right)$$

where a is the depth of *Fang* inserted into the steel sleeve, b is the thickness of the pressurized section of *Fang*, c is the angle formed by a and b, $\:{d}_{1}$ is the length of the upper pressurized zone, and $\:{d}_{2}$ is the length of the lower pressurized zone.

The volume of each part is calculated as:

7 $$\:{V}_{1}=\frac{1}{2}\bullet\:\left({d}_{1}\text{sin}\theta\:\right)\bullet\:\left({d}_{1}\text{cos}\theta\:\right)\bullet\:{h}_{l}$$

8 $$\:{V}_{2}={\int\:}_{0}^{1.5b}f\left(x\right)dx\bullet\:{h}_{l}$$

9 $$\:{V}_{3}=\frac{1}{2}\bullet\:\left({d}_{2}\text{sin}\theta\:\right)\bullet\:\left({d}_{2}\text{cos}\theta\:\right)\bullet\:{h}_{l}$$

According to Eqs. (3) and (8), the following can be obtained:

10 $$\:{V}_{2}={\int\:}_{0}^{1.5b}\delta\cdot{e}^{-\frac{1.5}{b}x}dx\cdot\:{h}_{l}$$

11 $$\:\delta\:={d}_{1}\bullet\:\text{sin}\theta\:$$

12 $$\:\Rightarrow\:{\:V}_{2}=\left({d}_{1}\text{sin}\theta\:\right)\bullet\:\left[\frac{b}{1.5}\bullet\:\left(1-{e}^{-\frac{9}{4}}\right)\right]\bullet\:{h}_{l}\approx\:\left({d}_{1}\text{sin}\theta\:\right)\bullet\:\left(0.6b\right)\bullet\:{h}_{l}$$

According to the equality of forces in the upper and lower compression zones, we can obtain:

13 $$\:{V}_{1}+{V}_{2}\approx\:{V}_{3}$$

14 $$\:\Rightarrow\:{{d}_{2}}^{2}=\frac{1.2{d}_{1}b}{\text{cos}\theta\:}+{{d}_{1}}^{2}$$

where $\:{h}_{l}$ is the height of the compressed section of *Fang.*

According to Hooke’s law^42^, the force $\:{F}_{1}$ at one end of the *Fang* to embed compression deformation is expressed as:

15 $$\:{F}_{1}=\frac{{E}_{T}\bullet\:{V}_{3}}{b}$$

According to small strain theory, $\:\text{sin}\theta\:\approx\:\theta\:$, $\:\text{cos}\theta\:\approx\:1$, and Eqs. (9) and (15) can be simplified as:

16 $$\:{F}_{1}=\frac{{E}_{T}}{b}\bullet\:\frac{1}{2}\bullet\:{{d}_{2}}^{2}{\bullet\:h}_{l}\frac{{\varDelta\:}_{c}}{\left(\frac{{l}_{f}}{2}-a\right)}$$

17 $$\:\Rightarrow\:{F}_{1}=\frac{{E}_{T}{{\bullet\:d}_{2}}^{2}{\bullet\:h}_{l}}{2b\bullet\:\left(\frac{{l}_{f}}{2}-a\right)}{\varDelta\:}_{c}$$

18 $$\:{k}_{c}=\frac{{E}_{T}{{\bullet\:d}_{2}}^{2}{\bullet\:h}_{l}}{2b\bullet\:\left(\frac{{l}_{f}}{2}-a\right)}$$

The parameter values that used for the calculation are $\:{E}_{T}$=159.9 MPa, a = 50 mm, b = 100 mm, $\:{h}_{l}$=150 mm, $\:{l}_{f}$=2000 mm. According to Eqs. (7), (9), (12), (14), (18), the value of the stiffness $\:{k}_{c}$ for the embedded deformation can be given as:

19 $$\:{k}_{c}=188.44\:N/mm$$

(2) Bending deformation stiffness.

In addition, *Fang* experienced bending deformation during the loading of the arch model. According to Fig. 22, the bending stiffness of the *Fang* was $\:{k}_{b}\:$calculated as:

20 $$\:{k}_{b}=\frac{48{E}_{WT}I}{{\left({l}_{f}-2\bullet\:a\right)}^{3}}$$

21 $$\:I=\frac{c\bullet\:{b}^{3}}{12}$$

where *I*, $\:{{k}_{b}}^{{\prime\:}}$ is the section moment of inertia of *Fang*, the stiffness provided by single *Fang*. And *c*,* b* are the height and width of the contact local section of *Fang*. The parameter values that used for the calculation are $\:{E}_{WT}$=7876.5 MPa, b = 100 mm, c = 50 mm, $\:{l}_{f}$=2000 mm. According to Eqs. (20), (21), the value of the bending stiffness can be obtained as:

22 $$\:{k}_{b}=229.67\:N/mm$$

(3) Overall stiffness of the Fang.

The deformation $\:{\varDelta\:}_{f}$ of a single *Fang* consists of embedded compression deformation $\:{\varDelta\:}_{c}$ and bending deformation $\:{\varDelta\:}_{b}$:

23 $$\:{\varDelta\:}_{f}={\varDelta\:}_{c}+{\varDelta\:}_{b}$$

24 $$\:\Rightarrow\:\frac{1}{{k}_{f}}=\frac{1}{{k}_{c}}+\frac{1}{{k}_{b}}$$

Thus the stiffness $\:{k}_{f}$ of a single *Fang* is calculated:

25 $$\:{k}_{f}=103.51\:N/mm$$

#### The stiffness provided by the beam

**Fig. 24:**
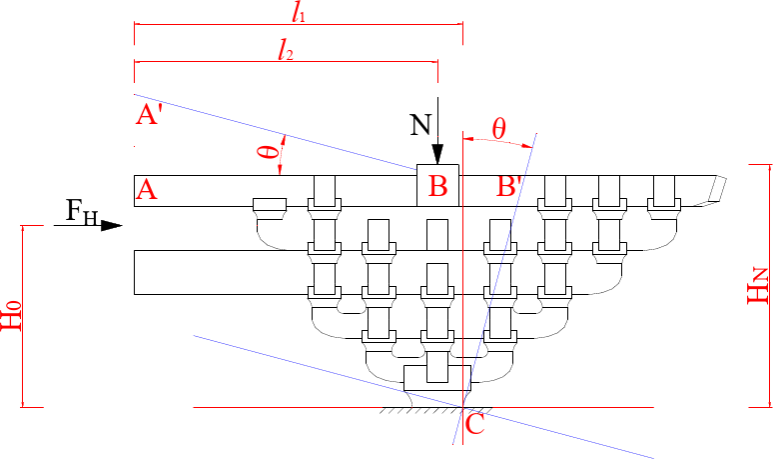
Main view of *Dou-Gong* under horizontal loading in Beam-direction.

Except for the constraints from *Fang*, the bottom *Lu-Dou* is extruded and deformed by the vertical force N. When the horizontal force *F* is loaded, the whole joint rotates at an angle of $\:\theta\:$ with point C as the fulcrum. Thus the stiffness provided by the beam is provided by both the rotation of the AB section beam and the overall rotation of the BC section as shown in Fig. 24.

(1) Beam rotational stiffness.

When the whole joint rotates at an angle of $\:\theta\:$, the vertical displacement of the end of the beam is $\:{l}_{2}$sin*θ*. The horizontal displacement of the loading segment is *H*sin, where *H* is the height of the beam subjected to the horizontal force and where $\:{l}_{2}$ is the length of the forced rotating beam section.

26 $$\:{k}_{l}^{{\prime\:}}=\frac{3{E}_{WR}{I}_{1}}{{{l}_{2}}^{3}}$$

27 $$\:{I}_{1}=\frac{{b}_{1}{{h}_{1}}^{3}}{12}$$

where $\:{k}_{l}^{{\prime\:}}$ is the vertical bending stiffness of each beam, $\:{E}_{WR}\:$is the R-direction bending modulus of elasticity, $\:{I}_{1}$ is the section moment of inertia of each beam, $\:{b}_{1}$, $\:{h}_{1}\:$are the section width and height of each beam.

By geometric analysis, the horizontal stiffness provided by each beam $\:{k}_{l}\:$is:

28 $$\:{k}_{l}=\frac{H}{{l}_{2}}{k}_{l}^{{\prime\:}}$$

The parameter values used for the calculation are shown in Table 5, where the numbering is from Fig. 3.

**Table 5:** Parameters related to the beam rotational stiffness.

Modulus	Explanation	Unit	value
$\:{E}_{WR}$	R-direction bending modulus of elasticity	MPa	8153.5
$\:{b}_{1}$ (C1)	Beam C1-section width	mm	100
$\:{h}_{1}$ (C1)	Beam C1-section height	mm	210
H (C1)	Beam C1-height of force point	mm	640
$\:{b}_{1}$ (C2)	Beam C2-section width	mm	100
$\:{h}_{1}$ (C2)	Beam C2-section height	mm	150
H (C2)	Beam C2-height of force point	mm	1030
$\:{l}_{2}$	Beam forced bending length	mm	1550

Therefore the beam rotational stiffness is calculated as:

29 $$\:{k}_{l}=332.08 N/mm$$

(2) Lu-Dou rotational stiffness.

**Fig. 25:**
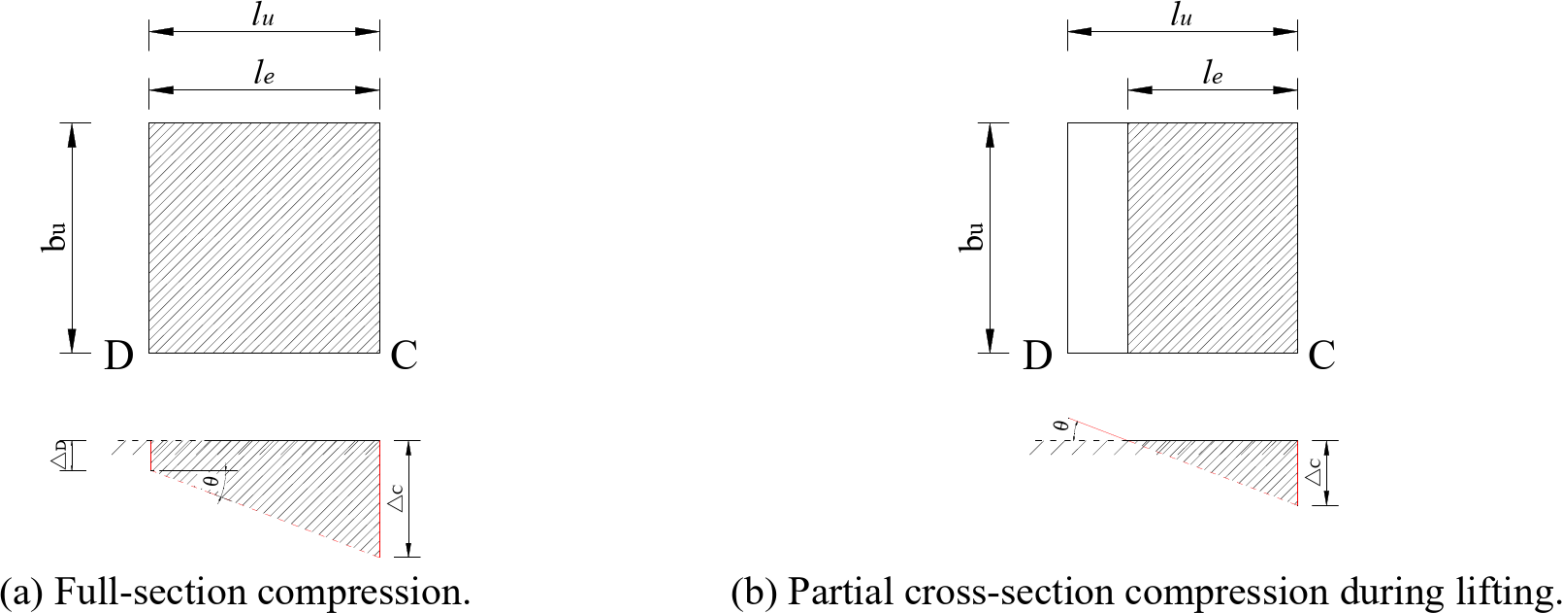
*Lu-Dou* bottom surface force state.

As shown in Fig. 24, the wooden components within the vertical compression range of *Lu-Dou*under vertical force are considered as virtual columns^33,43^, and the lateral inclination angle $\:\theta\:\:$occurs under the action of vertical force *N* and horizontal force *F*. The force state of the bottom of *Lu-Dou* is shown in Fig. 25. Since the initial stiffness is studied in this paper, only the full-section compression stage is considered.

30 $$\:\text{Tan}\theta\:=\frac{{\varDelta\:}_{C}-{\varDelta\:}_{D}}{{l}_{u}}$$

31 $$\:\text{Tan}\theta\:=\frac{{\varDelta\:}_{L}}{{H}_{N}}$$

where $\:{\varDelta\:}_{C}$, $\:{\varDelta\:}_{D}$ is the deformation at points C, D, $\:{l}_{u}$ is the length of the bottom surface of *Lu-Dou*, and $\:{H}_{N}$ is the height of the vertical force application point, $\:{\varDelta\:}_{L}$ is the horizontal displacement of *Lu-Dou.*

32 $$\:{\varDelta\:}_{C}=\frac{{\sigma\:}_{C}}{{E}_{R}}\bullet\:{H}_{N};{\:\varDelta\:}_{D}=\frac{{\sigma\:}_{D}}{{E}_{R}}\bullet\:{H}_{N}$$

33 $$\:{\sigma\:}_{C}=\frac{N}{{S}_{0}}+\frac{{F}_{0}\bullet\:{H}_{0}+N\bullet\:{\varDelta\:}_{L}}{W};\:{\sigma\:}_{D}=\frac{N}{{S}_{0}}-\frac{{F}_{0}\bullet\:{H}_{0}+N\bullet\:{\varDelta\:}_{L}}{W}$$

34 $$\:W=\frac{1}{6}\bullet\:{b}_{u}\bullet\:{{l}_{u}}^{2}$$

where $\:{\sigma\:}_{C}$, $\:{\sigma\:}_{D}$ is the stress at points C, D, $\:{S}_{0}$ is the area of the compression zone, *W* is the sectional resistance moment, $\:{E}_{R}$ is R-direction compressive modulus of elasticity, $\:{F}_{0}$ is the horizontal combined force, $\:{H}_{0}$ is the height of action of the horizontal combined force, $\:{b}_{u}$ is the width of the bottom surface of *Lu-Dou.*

According to Eqs. (30)-(34), it can be obtained that:

35 $$\:{F}_{0}=\frac{{l}_{u}\bullet\:{E}_{R}\bullet\:W-2 N\bullet\:{{H}_{N}}^{2}}{2{{H}_{N}}^{2}\bullet\:{H}_{0}}\bullet\:{\varDelta\:}_{L}$$

36 $$\:{k}_{u}=\frac{{l}_{u}\bullet\:{E}_{R}\bullet\:W-2 N\bullet\:{{H}_{N}}^{2}}{2{{H}_{N}}^{2}\bullet\:{H}_{0}}$$

The value of Lu-Dou rotational stiffness $\:{k}_{u}$ calculated as:

37 $$\:{k}_{u}=5.07 N/mm$$

(3) Overall stiffness of the beam.

Since the overall stiffness comes from the beam rotational stiffness and the *Lu-Dou* rotational stiffness, combining the Eqs. (29) and (37), it can be concluded that the horizontal stiffness *K*_*B*_ of *Dou-Gong* in Beam-direction is:

38 $$\:{k}_{m}={k}_{l}+{k}_{u}=337.15 N/mm$$

*Total stiffness in Beam-direction*.

Combining Eqs. (25) and (38), the total stiffness in Beam-direction $\:{K}_{T}$:

39 $$\:{K}_{T}=n\bullet\:{k}_{f}+{k}_{m}$$

where *n* is the number of ties to Fang. According to Table 3, the difference between the formula and experimental values for loading in Beam-direction can be obtained in Table 6.

**Table 6:** Comparison table of formula calculation results and experiment results in Beam-direction.

	Dou-Gong	Experiment results (*N*/mm)	Formula result (*N*/mm)	The error value
Beam-direction	DG1	412.1	440.66	6.93%
	DG2	575	647.68	12.64%
	DG3	781.3	854.70	9.39%

Stiffness formula in *Fang*-direction.

**Fig. 26:**
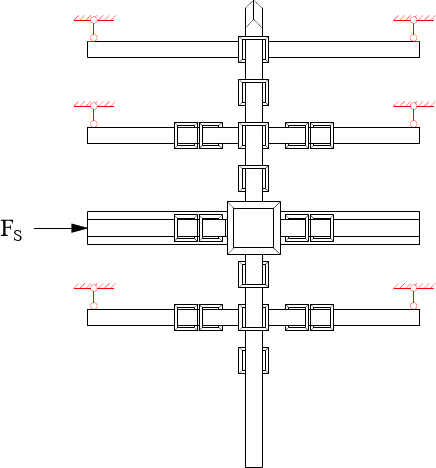
Bottom view of *Dou-Gong* under horizontal loading in *Fang*-direction.

When the horizontal load acted in *Fang*-direction, The beams were not restrained, and the *Fei-Qi-Xin-Fang* was supported only by steel sleeves and was not restrained in displacement as shown in Fig. 26.

**Fig. 27:**
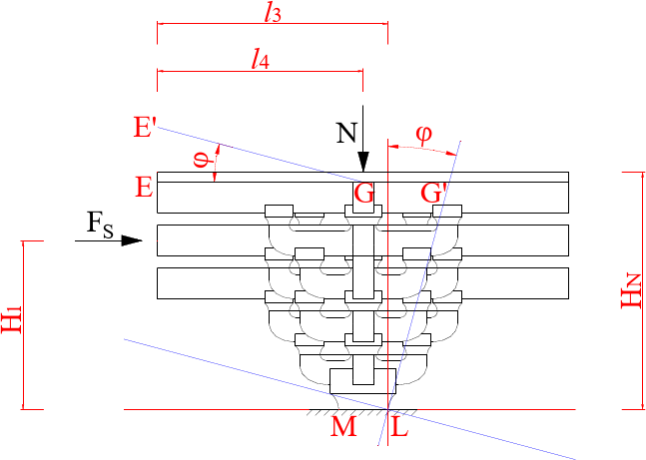
Sid view of *Dou-Gong* under horizontal loading in *Fang*-direction.

Consistent with Fig. 24, when the horizontal force $\:{F}_{1}$ is loaded, the whole joint rotates at an angle of $\:\phi\:$ with point C as the fulcrum. Thus the stiffness provided by the beam is provided by both the rotation of the EG section beam and the overall rotation of the LG section as shown in Fig. 27.

According to Eqs. (26), (27), (28), (36), the total horizontal stiffness of *Dou-Gong* in *Fang*-direction is:

40 $$\:{K}_{S}={k}_{l1}+{k}_{u1}$$

41 $$\:{k}_{l1}=\frac{{E}_{WR}}{4{{l}_{4}}^{4}}\bullet\:\sum\:_{i=1}^{3}{H}_{1i}\bullet\:{b}_{1i}\bullet\:{{h}_{1i}}^{3}$$

42 $$\:{k}_{u1}=\frac{{E}_{R}\bullet\:{{l}_{u}}^{2}\bullet\:{{b}_{u}}^{2}-12 N\bullet\:{{H}_{N}}^{2}}{12{{H}_{N}}^{2}\bullet\:{H}_{1}}$$

where $\:{K}_{S}$ is the stiffness formula in Fang-direction, $\:{k}_{l1}$ is the horizontal stiffness provided by *Qi-Xin-Fang*, and $\:{k}_{u1}$ is the *Lu-Dou* rotational stiffness around the L point. The parameter values that used for the calculation are shown in Table 7.

**Table 7:** Parameters related to the stiffness formula in Fang-direction.

Modulus	Explanation	Unit	value
$\:{b}_{11}$ (A1)	*Qi-Xin-Fang* A1-section width	mm	100
$\:{h}_{11}$ (A1)	*Qi-Xin-Fang* A1-section height	mm	100
$\:{H}_{11}$ (A1)	*Qi-Xin-Fang* A1-height of force point	mm	615
$\:{b}_{12}$ (A2)	*Qi-Xin-Fang* A2-section width	mm	100
$\:{h}_{12}$ (A2)	*Qi-Xin-Fang* A2-section height	mm	100
$\:{H}_{12}$ (A2)	*Qi-Xin-Fang* A2-height of force point	mm	825
$\:{b}_{13}$ (A3)	*Qi-Xin-Fang* A3-section width	mm	200
$\:{h}_{13}$ (A3)	*Qi-Xin-Fang* A3-section height	mm	100
$\:{H}_{13}$ (A3)	*Qi-Xin-Fang* A3-height of force point	mm	1060
$\:{l}_{4}$	*Qi-Xin-Fang* forced bending length	mm	1100
$\:{H}_{1}$	Height of the horizontal combined force	mm	825

As seen from Tables 6 and 8, the error between the value obtained via the calculation formula of horizontal stiffness and the experimental result is less than 15%, and most of them are less than 10%. In conclusion, the formulas for the horizontal stiffness of *Dou-Gong* are effective and reliable.

**Table 8:** Comparison table of formula calculation results and experiment results.

	Dou-Gong	Experiment results (*N*/mm)	Formula result (*N*/mm)	The error value
Fang-direction	DG1	532.2	502.03	−5.67%
	DG2	477.7	502.03	5.09%
	DG3	550.8	502.03	−8.86%

## Conclusions

In this study, the mechanical properties from two different loading directions in *Dou-Gong* of Song dynasty were studied through experimental and theoretical methods, and several important conclusions can be drawn:

(1) Under the action of horizontal low-cycle loading, the whole *Dou-Gong* exhibits a good seismic performance. The influence of the number of *Fang*s on the overall mechanical properties of the bucket is more obvious when loaded in Beam-direction, which is embodied in that *Dou-Gong* with a small number of *Fang* end the test with failure, while the joints with a large number of *Fang*s end with excessive displacement, the overall failure presents obvious brittle failure characteristics. However, the relationship between the number of *Fang*s and the overall displacement of the structure is not obvious when loaded in *Fang*-direction. The three joints all end the test with too much displacement, and the strength degradation coefficient of each level of *Dou-Gong* is generally greater than 0.9, indicating ductile failure.

(2) In Beam-direction, the bearing capacity of *Dou-gong* increases continuously with the increase of horizontal displacement. After *Fang* is damaged, however, the bearing capacity starts to decrease. The bearing capacity and the stiffness of *Dou-Gong* increase with increasing number of *Fang*. And the stiffness of *Dou-Gong* increases by about 29% with each added connecting *Fang*. In *Fang*-direction, *Dou-Gong* stiffness is not greatly affected by the number of *Fang*. The positive horizontal bearing capacity increases slightly as the number of *Fang* rises, while the negative horizontal bearing capacity shows a slight decrease. This range of influence remains within ± 10%.

(3) When the loading direction is the same, the deformation capacity of *Dou-Gong* decreases with increasing number of connecting *Fang*. For every additional set of *Fang*, the plastic deformation ratio decreases by about 5 −10%. The plastic deformation in *Fang*-direction is about 10% greater than that in Beam-direction.

(4) Through the analysis of the mechanical properties of *Dou-Gong*, the calculation formulas of the stiffness in Beam-direction and *Fang*-direction in the elastic stage are obtained. The two dimensional stiffness calculation formula of *Dou-Gong* can be used in the analysis and calculation of the mechanical properties of *Dou-Gong* in the future.

(5) Through experiments and theoretical analysis, the stiffness in Beam-direction is determined to be due to two main aspects: the stiffness provided by *Fei-Qi-Xin-Fang* and the stiffness provided by beams. The stiffness in *Fang*-direction mainly comes from the stiffness provided by the *Qi-Xin-Fang*. The *Lu-Dou* rotational stiffness has little effect on the lateral stiffness of the elastic stage of *Dou-Gong*, within 10%.

## Data Availability

The data used to support this study are included within the article.
